# Machine Learning and Infrared Thermography for Fiber Orientation Assessment on Randomly-Oriented Strands Parts

**DOI:** 10.3390/s18010288

**Published:** 2018-01-19

**Authors:** Henrique Fernandes, Hai Zhang, Alisson Figueiredo, Fernando Malheiros, Luis Henrique Ignacio, Stefano Sfarra, Clemente Ibarra-Castanedo, Gilmar Guimaraes, Xavier Maldague

**Affiliations:** 1 School of Computer Science, Federal University of Uberlandia, Uberlandia 38408-100, Brazil; 2 Department of Electrical and Computer Engineering, Computer Vision and Systems Laboratory (CVSL), Laval University, Quebec City, QC G1V 0A6, Canada; hai.zhang.1@ulaval.ca (H.Z.); clemente.ibarra-castanedo@gel.ulaval.ca (C.I.-C.); Xavier.Maldague@gel.ulaval.ca (X.M.); 3 Department of Mechanical Engineering, Laboratory of Teaching and Researching on Heat Transfer, Federal University of Uberlandia, Uberlandia 38408-100, Brazil; alissonfigueiredo@hotmail.com.br (A.F.); fernandomalheiros@gmail.com (F.M.); luishenrique.meta@yahoo.com.br (L.H.I.); gguima@ufu.br (G.G.); 4 Department of Industrial and Information Engineering and Economics, University of L’Aquila, Roio Poggio, L’Aquila (AQ) 67100, Italy; stefano.sfarra@univaq.it

**Keywords:** infrared thermography, flying laser spot, fiber orientation, randomly-oriented strands

## Abstract

The use of fiber reinforced materials such as randomly-oriented strands has grown in recent years, especially for manufacturing of aerospace composite structures. This growth is mainly due to their advantageous properties: they are lighter and more resistant to corrosion when compared to metals and are more easily shaped than continuous fiber composites. The resistance and stiffness of these materials are directly related to their fiber orientation. Thus, efficient approaches to assess their fiber orientation are in demand. In this paper, a non-destructive evaluation method is applied to assess the fiber orientation on laminates reinforced with randomly-oriented strands. More specifically, a method called pulsed thermal ellipsometry combined with an artificial neural network, a machine learning technique, is used in order to estimate the fiber orientation on the surface of inspected parts. Results showed that the method can be potentially used to inspect large areas with good accuracy and speed.

## 1. Introduction

The use of fiber reinforced composite materials in astronautics, aeronautics, automotive, wind energy, oil and gas and petrochemical industries is vital for the full development of these key areas for economic and social prosperity of a country mainly because of composite material’s unique and advantageous properties: they are typically lighter and more resistant to corrosion than the metallic materials traditionally used in these industries.

Composite materials (CM) in transport aircraft components have been used for decades. Prior to the mid-1980s, aircraft manufacturers used CM in transport category aircraft in secondary structures (e.g., wing edges) and control surfaces. In 1988, Airbus introduced the A320, the first aircraft in production with an all-composite tail section and, in 1995, the Boeing Company introduced the Boeing 777, also with a composite tail section. In recent years, manufacturers have expanded the use of composites to the fuselage and wings. CM used in commercial aircraft are typically produced by combining layers of continuous carbon or glass long-fibers with epoxy.

The replacement of metal alloys by polymeric composites, besides providing a reduction of aircraft weight of 20% to 30% (and, consequently, a payload increase—both passengers and transported goods), and fuel economy, it also increases flight autonomy, i.e., for how long the aircraft can operate without refueling, and decreases the final cost of components up to 25% [1]. Another example is the *C Series* aircraft from Bombardier, which contains 70% advanced materials comprising 46% composite materials and 24% aluminium-lithium representing 15% lower seat-mile cost and a significant reduction in maintenance costs.

The use of composite materials has grown during the past years, and this growth is expected to continue in the near future. The aeronautic industry uses composite material intensively. Techniques and methodologies to ensure the quality of parts, components and structures made with such class of advanced materials, both in the manufacturing stage and throughout its lifetime, are indispensable. Therefore, Non-Destructive Evaluation (NDE) methods today play a fundamental role. NDE regroups a variety of methods used to examine or inspect a part or a system without impairing its future usefulness. Several studies have recently investigated the pros and cons of such techniques in a variety of applications [2,3,4,5].

Infrared thermography (IRT) is a safe NDE technique that has a fast inspection rate and is generally contactless. It is used for diagnostics and monitoring in several fields such as electrical components, thermal comfort, buildings, artworks, composite materials and others. IRT popularity has grown in recent years due to spatial resolution and acquisition rate improvements of infrared (IR) cameras while becoming more affordable. Another factor contributing to IRT’s popularity is the development of advanced image processing techniques that improve defect detection and characterization. In active IRT, an external heat source is used to stimulate the material being inspected in order to generate a thermal contrast between the feature of interest and the background. The active approach is adopted in many cases given that the inspected parts are usually in equilibrium with the surroundings and an external heat source is required to provoke a thermal contrast between the non-defective and defective areas [6].

The arrangement or orientation of the fibers relative to one another, the fiber concentration, and the distribution all have a significant influence on the strength and other properties of fiber reinforced composites. Thus, effective testing techniques are needed to assess fiber content. Destructive methods can be employed to evaluate a composite fiber content, e.g., cutting a section of the material, polishing the area and evaluating it by microscopy. However, the destructive approach is not always an option, since the sample will be “damaged” after the inspection and probably unfit for use. Thus, NDE techniques must be employed in some cases to assess the material’s fiber content.

In this paper, we present a technique to assess the fiber orientaion on fiber reinforced materials by means of IRT. Tests were performed on different carbon fiber reinforced polymer samples (CFRP). This technique is called Pulsed Thermal Ellipsometry (PTE). Two different approaches are exploited in this paper. First, the traditional point heating source inspection approach, based on laser spot thermography technique (LST), is reviewed. Then, a method is proposed, which uses a line heating approach, based on laser line thermography technique (LLT), combined with an artificial neural network (ANN), which is a machine learning technique, for the inspection of large areas in short times. This technique is specially suitable for the case of randomly oriented strand (ROS) samples which have random fiber orientation. Finally, experimental results are presented and discussed for both methods.

## 2. Methodology

### 2.1. Pulsed Thermal Ellipsometry

#### 2.1.1. Point Heating Source Approach

More than one century ago, De Senarmont [7] applied a thermal approach to find out the principal orientations in crystal plates: he covered them with a thin layer of wax, heated them over a small spot and monitored the isotherm shape revealed by the solid/liquid transition contour appearing in the wax layer. The isotherm proved to be elliptical and its aspect ratio was found to be related to the square root of the principal conductivities in the surface plane.

This method, referred by Krapez [8] as Thermal Ellipsometry (TE), was later used, with, of course, up-to-date experimental equipment, in several applications by the means of IRT [8,9,10,11,12,13,14,15]. It was applied on polymer materials to establish a correlation between their draw ratio and the induced thermal anisotropy. It was also used to evaluate the fiber orientation in the case of composite materials using short or long carbon fibers. In this work, the method of evaluating the fiber orientation in the case of CMs is referred as PTE.

**Figure 1:**
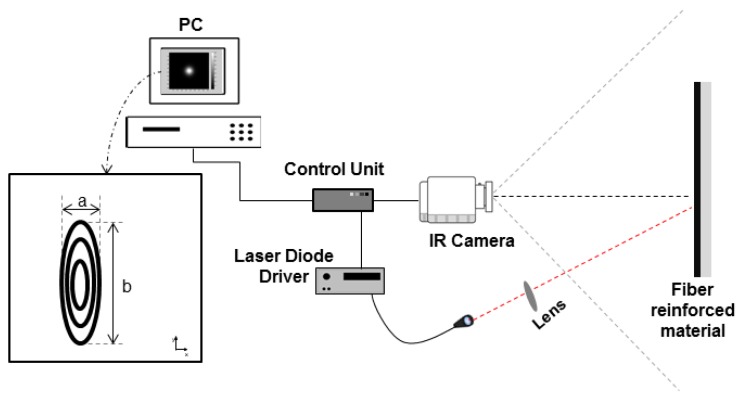
Pulsed Thermal Ellipsometry (PTE) set-up. Adapted from [16].

PTE, which is a LST method, is an inspection method that enables the assessment of fiber orientation on CM. It involves the spot heating of the sample’s surface and following the observation of the heated pattern with an infrared camera. A short pulse is used to spot heat the sample. If the material has an oriented structure, such as CFRP, an elliptical thermal pattern is observed, with the ratio between the two principal axes (b/a) being related to the square root of the thermal diffusivities in the longitudinal and transverse directions. A test on an isotropic material would produce a circle instead of an ellipse. Figure 1 shows a typical PTE experimental set-up.

The ellipse’s major axis, b, has the same orientation than the fibers on that region. This “elliptical” behaviour occurs due to the difference in the thermal conductivities values on the surface of the sample. The thermal conductivity value parallel to the fibers is greater than the value perpendicular to them, i.e., material is thermally anisotropic: $k_{∥}>k_{⊥}$. Thus, heat will travel faster on the direction parallel to the fibers and consequently the thermal pattern observed on the surface will be larger on the direction of $k_{∥}$, which results in an elliptical pattern. The heat diffusion process is recorded with an IR camera and stored in a 3D matrix (or an array of images). To extract the elliptical pattern from the IR images, i.e., thermograms, some image processing techniques must be applied. This approach is fully presented in a previous work from the authors [16]. In [16], PTE was successfully applied for laminates with continuous fibers. In this kind of materials, fiber orientation is homogeneous. Thus, one single point heating source inspection is sufficient to assess the material’s fiber orientation. On the other hand, for ROS samples, where fiber orientation is randomly distributed on the material’s surface, would need to be inspected at several points in order to have a good assessment of the materials fiber orientation and distribution. Consequently, such approach would prove very time consuming and unfit for industrial application. Next, a line heating approach is presented for fiber orientation assessment which makes possible the inspection of larger areas at once.

#### 2.1.2. Line Approach

A second inspection approach was used to produce a heating line on the surface of samples. It is based on a dynamic point scanning approach which is similar to a LLT method. The use of a flying laser spot (FLS) technique in combination with an ANN was proposed in our previous work [17] to assess the fiber orientation over a line region on the surface of a ROS plate. In this paper, we conduct a brief review of the approach proposed in [17] and present additional and unpublished results.

FLS is a dynamic active IRT technique where the inspected sample and the heating source are in relative motion. This can be done in two ways, the IR camera and an heating source move along the surface while the sample being inspected is motionless, or it may be the sample that moves while the camera and heating source are motionless. In both cases, the thermal history for every pixel can be precisely tracked by controlling the displacement speed and the rate of data acquisition. Detailed theoretical and experimental aspects of this technique can be found in [18].

**Figure 2:**
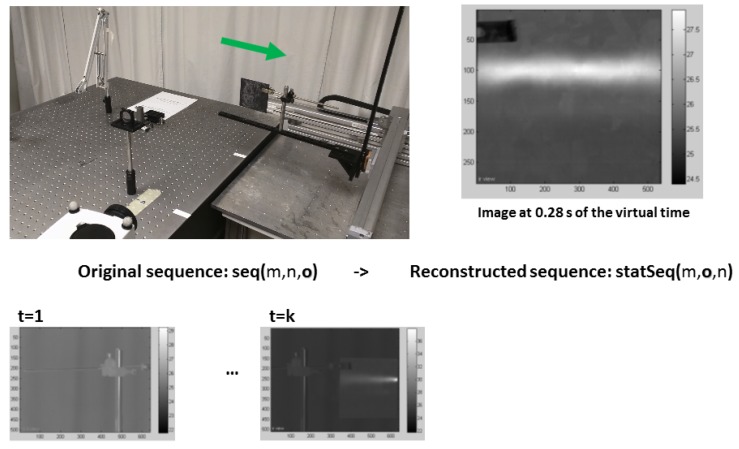
Pseudo-static sequence reconstruction. At the bottom of the figure, two images from the original sequence at different times are shown. Above these images, the experimental set-up is shown: the IR camera and the inspected sample can be seen as well as the 2-axis electric actuator used to displace the sample. The green arrow indicates the direction of displacement. On the right, an image from the reconstructed pseudo-static sequence is shown. Adapted from [17].

In this paper, as it was applied in [17], the second displacement approach is adopted, i.e., the sample moves in front of the camera. This choice was made due to experimental set-up restrictions. For each inspection, the sample moves (horizontally from left to right in reference to Figure 2) in front of the camera and the laser beam. This movement of the sample is performed by a 2-axis electric actuator that moves the inspected sample in front of the camera’s field of view enabling to control the inspection speed displacement and acquisition rate in a precise manner.

In the recorded image sequence, the sample appears to be moving. To apply any image process technique, the images must be rearranged into a pseudo-static set of images so that the sample appears to be standing still and consequently traditional IRT data processing techniques could be applied similarly to the data obtained with static IRT inspection techniques such as pulsed thermography (PT). As described in [17], the reconstructed sequence is obtained by following the temporal evolution of every pixel independently, in such a way that, a given pixel of the original sequence $P(x_{i},y_{j},t)$, is recovered frame by frame through time *t* and reallocated into a new image. For instance, a pixel *P* that is in a given position at time *t* will be in a different position at a later time. Figure 2 shows some images of an original sequence and an image from the corresponding pseudo-static reconstructed sequence 0.37 s after the pulse. Part of the experimental set-up used is also shown (the laser source is not shown). The arrow indicates the direction of the sample’s movement. A detailed procedure on how to reconstruct the dynamic sequence in order to obtain a pseudo-static sequence is provided in [19].

After the pseudo-static sequence is obtained, some advanced infrared image processing technique must be applied in the reconstructed sequence in order to perform some features selection to be used in the machine learning step. In this work, three processing techniques were tested: dynamic thermal tomography (DTT) [20], pulsed phase thermography (PPT) [21] and principal component thermography (PCT) [22]. Next, these techniques are briefly described.

### 2.2. Image Processing Techniques

#### 2.2.1. Dynamic Thermal Tomography (DTT)

Dynamic Thermal Tomography , originally proposed by [20], pixel temperature profiles are rearranged so the time where the maximum temperature occurred for each pixel can be displayed in a single image. This rearranged would depict some of the most important information regarding the thermal diffusion inside the solid which is its maximum. With one compare all these maximum times together, it could lead to valuable information concerning the sample’s structure.

#### 2.2.2. Pulsed Phase Thermography (PPT)

In PPT, originally proposed by [21] and recently reviewed by [23], data are transformed from the time domain to the frequency spectra using the 1D DFT (Discrete Fourier transform):

(1) $$F_{n}=\Delta t\sum_{k=0}^{N-1}T\left(k\Delta t\right)exp^{\left(-j2\pi nk/N\right)}=Re_{n}+Im_{n}$$

where *j* is the imaginary number, *n* designates the frequency increment (n=0,1,…,N), Δt is the sampling increment, and Re and Im are the real and imaginary parts of the transform, respectively. In this case, real and imaginary parts of the complex transform are used to estimate the amplitude and the phase as described in [24]:

(2) $$A_{n}=\sqrt{Re_{n}^{2}+Im_{n}^{2}}$$

(3) $$\phi_{n}=tan^{-1}\left(\frac{Im_{n}}{Re_{n}}\right)$$

DFT can be applied to any waveform. The phase, Equation (3), is of particular interest in NDE given that it is less affected than raw thermal data by environmental reflections, emissivity variations, non-uniform heating, and surface geometry and orientation. These phase characteristics are very attractive not only for qualitative inspections but also for quantitative characterization of materials as will be pointed out latter.

As any other thermographic technique, PPT is not without drawbacks. The noise content is considerable, especially at high frequencies. A de-noising step is therefore often required. A combination of PPT and thermographic signal reconstruction (TSR) [25] is an interesting possibility, reducing noise and allowing the depth retrieval of defects as proposed in [26]. A linear relationship exists between defect depth *z* and the inverse square root of the blind frequency (which can be observed from experimental data). According to [27], blind frequency ($f_{b}$) is the frequency at which the phase contrast is enough for a defect to be visible (at frequencies higher than $f_{b}$, it is not possible to detect it). The thermal diffusion length (μ), described by [28], can be used to fit experimental data and estimate the depth (*z*) as proposed by [26]:

(4) $$z=C_{1}\sqrt{\frac{\alpha }{\pi \times f_{b}}}=C_{1}\mu$$

where the thermal diffusion length is $\mu =\left(2\alpha /\omega \right)$ (m), ω (rad/s) is the angular frequency, α is the thermal diffusivity of the material, $f_{b}$ (Hz) is the blind frequency and $C_{1}$ is an empirical constant where has been observed that $1.5<C_{1}<2$ when working with the phase, with $C_{1}=1.82$ typically adopted in several work such as in [29,30].

#### 2.2.3. Principal Component Thermography (PCT)

Principal Component Thermography, originally proposed by [22], extracts the image features and reduces the undesirable signals. It relies on Singular Value Decomposition (SVD), which is a tool to extract spatial and temporal data from a matrix in a compact manner by projecting original data onto a system of orthogonal components known as Empirical Orthogonal Functions (EOF). By sorting the principal components in such way that the first EOF represents the most characteristic variability of the data, the second EOF contains the second most important variability, and so on, the original data can be adequately represented with only a few EOFs.

The SVD of a M×N matrix A, where M>N, can be calculated as follows:

(5) $$A=URV^{T}$$

where *U* is a M×N orthogonal matrix, *R* being a diagonal M×N matrix (with singular values of *A* present in the diagonal), $V^{T}$ is the transpose of a M×N orthogonal matrix (characteristic time) as proposed in [22].

Hence, to apply the SVD to thermographic data, the 3D thermogram matrix representing time and spatial variations has to be reorganized as a 2D M×N matrix *A*. This can be done by rearranging the thermograms for every time as columns in *A*, in such a way that time variations will occur column-wise while spatial variations will occur row-wise. Under this configuration, the columns of *U* represent a set of orthogonal statistical modes known as EOF that describe the data spatial variations. On the other hand, the principal components (PCs), which represent time variations, are arranged row-wise in matrix $V^{T}$. The first EOF will represent the most characteristic variability of the data; the second EOF will contain the second most important variability, and so on. Usually, original data can be adequately represented with only a few EOFs. Typically, an IR sequence of 1000 images can be replaced by 10 or less EOFs.

### 2.3. Artificial Neural Network (ANN)

An ANN is a biologically-inspired programming paradigm which enables a computer to learn from observational data. It is composed of a large number of highly interconnected processing elements (neurons) working together to solve specific problems. ANNs, like people, learn by example. An ANN is configured for a specific application, such as pattern recognition or data classification, through a learning process which could be supervised or unsupervised. Learning in artificial networks, like in biological systems, involves adjustments to the synaptic connections that exist between the neurons. There are several works involving ANN in the literature and a review on image processing with ANN can be found in [31]. In NDE, machine learning techniques such as ANN have been used for years in application involving coat thickness prediction and defect depth estimation [32,33,34].

In this work, the thermal pattern obtained from the pseudo-static sequence is divided into small sections (or samples) that represent different points on the inspected line. A multilayer perceptron (MLP) feed-forward network, with sigmoid hidden and *softmax* output neurons (512 and 4, respectively) trained with scaled conjugate gradient backpropagation is then used to classify these points on the reconstructed line obtained with the pseudo-static IR sequence into their corresponding class (in this case: fiber orientation) as it was proposed in [17]. The points (samples) extracted from the pseudo-static line are divided into three sets of samples: 70% of samples is used to train the network, 15% is used for validation during training and the remaining 15% is used for testing the network after the training has been completed. Next, data representation of input samples is detailed as well as how classes are organized.

#### 2.3.1. ANN Input

**Figure 3:**
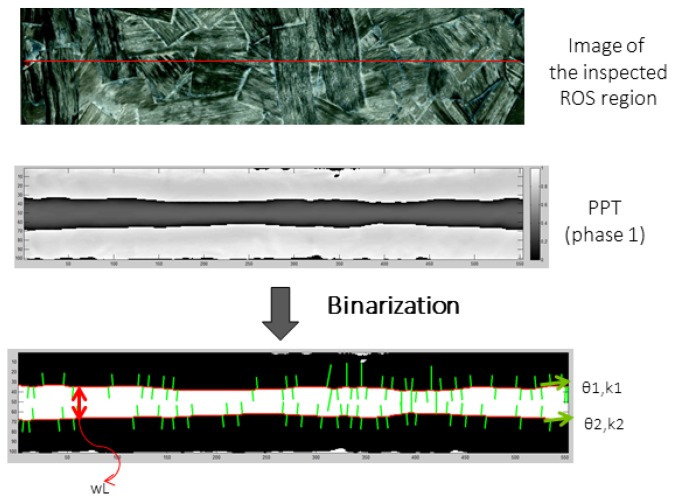
Summary of the process to obtain the envelope line, from where the features used in the Artificial Neural Network (ANN) are selected, from a Flying Laser Spot (FLS) inspection of a Randomly Oriented Strand (ROS) sample.

**Figure 4:**
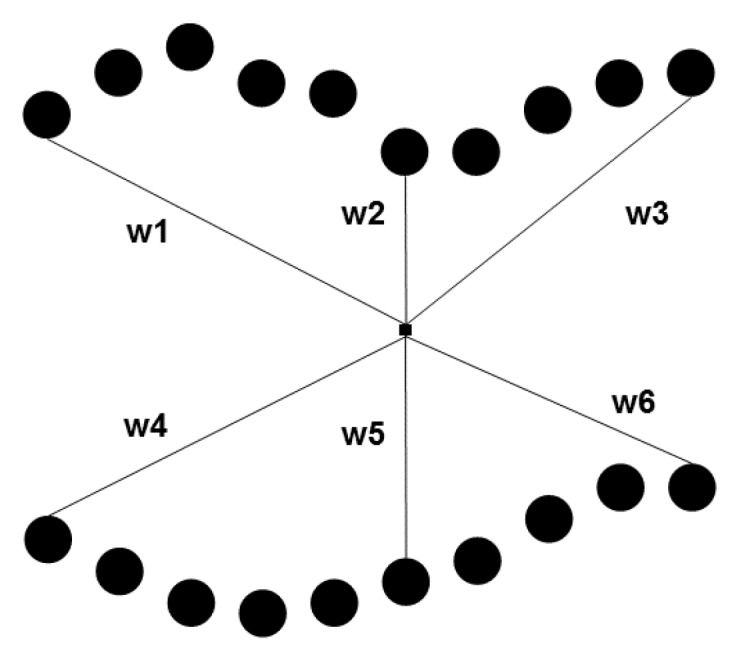
Line envelope width of a single sample. w1 … w6 are the distance between the sample’s centroid and six points on the envelope.

After the thermal pattern obtained with the line approach (FLS) is reconstructed into the pseudo-static sequence, the resulting image sequence is processed with one of the three techniques mentioned before: PCT, PPT or DTT. Then, the processed image is binarized using an automatic threshold selection based on the Otsu’s method. The envelope of the binary line image is easily selected and then 11 features are calculated for each sample. Figure 3 presents an example of the envelope extraction from the binary image. Each sample has two line segments originating from the line envelope: top and bottom line segments. These line segments belong to the edges of the binary image. The first two extracted features are the normal to the line segment orientation regarding the x-axis of the top and bottom segments: $\theta_{1}$ and $\theta_{2}$, respectively. The next two features are the curvature values ($k_{1}$ and $k_{2}$: it is a measure of how much the curve deviates from a straight line) in the middle points of the top and bottom line segments. The fifth feature is the width of the envelope on its middle section, wL. The last six extracted features are also related to the width of the envelop: the distance (in pixels) from the envelope’s centroid to six points on the envelope edges (Figure 4 shows the position of these points).

The processed pseudo-static image is divided in the same manner that the binary image. Then, the pixels of each sample are rearranged line-wise. The input data of the ANN, i.e., the data used to describe each sample, are the combination of the 11 features calculated before, with the line-wise pixels from the EOF image and its immediate left and right neighbors. In total, each sample is represented by a set of 2681 numerical values. These values are the input of the network.

#### 2.3.2. ANN Output

**Figure 5:**
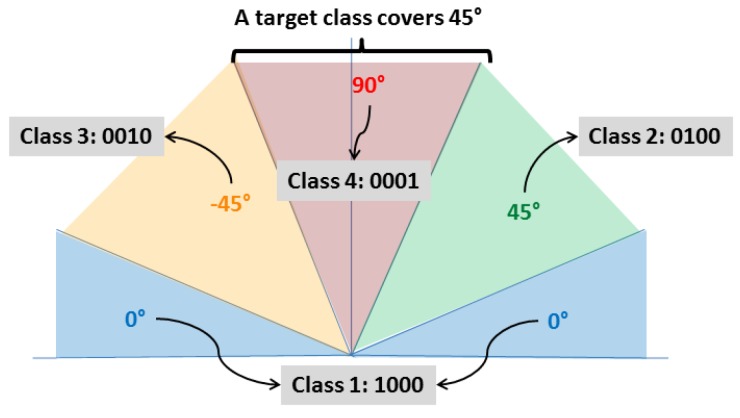
How recognition classes are divided. Each class covers a range of 45°. Adapted from [17].

The output of the ANN is the class of the sample presented in the input layer. In this work, samples were classified into four classes. Each class covers a range of 45°. For instance, class 4 is centered at 90° and represents orientation angles ranging from 67.5° to 112.5°. Figure 5 shows an schematic example explaining how the four classes are divided. Thus, the output layer of the ANN has four neurons. Each neuron is in charge of recognizing one class, i.e., if the sample presented in the input layer belongs to the first class the first neuron would has the value 1 and the other neurons value would be 0 (in the perfect recognition scenario).

#### 2.3.3. ANN Training

**Figure 6:**
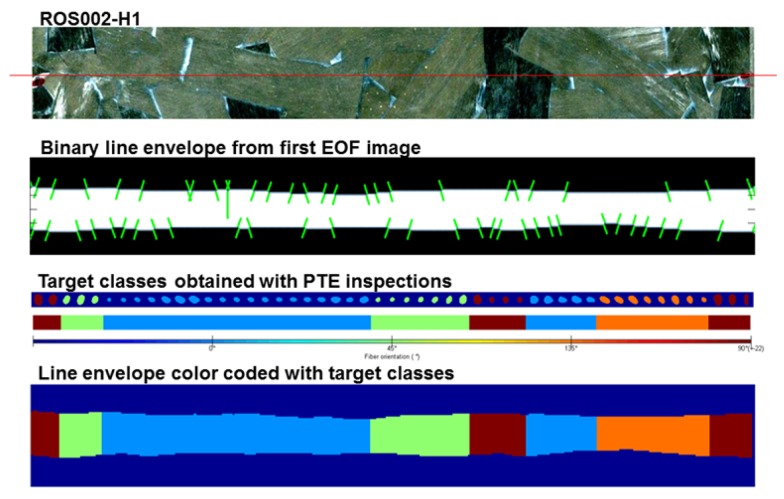
Class assignment of a line inspection. The line was divided into 49 samples and for each one of this samples a PTE spot inspection was performed in order to define the individual’s class. e.g., the PTE inspection assessed a fiber orientation of 16°, the class of this particular individual would be 0 (or Class 1000 according to the output neurons). The colors considered here are the same as the ones applied in Figure 5.

To train the network, target classes were previously determined by PTE static-point inspections. For each one of the 49 samples on the line, a respective PTE inspection was previously performed, i.e., a point-by-point inspection: for each line, 49 samples, with 2 mm spacing, were inspected with the PTE approach proposed in [16]. Then, based on the orientation angle obtained with the PTE inspection, a target class was assigned to the sample. An example of this target association with the 49 samples of a line can be found in Figure 6. All 180 angle degrees possibilities were not considered as individual classes because it would make the classification processes impossible due to the lack of information present on the line envelope that could be extracted. For instance, the distinction between an input belonging to class 15° and an input belonging to class 16° would be impossible. In total, 12 lines, from three different samples (ROS001, ROS002 and ROS003) were inspected and the population was formed by 588 individuals.

## 3. Results and Discussion

### 3.1. Pulsed Thermal Ellipsometry Results

To evaluate the effectiveness of the PTE approach for ROS samples, individual strands were first inspected separately. A single spot was heated on the surface of the sample in a predefined position for 0.1 s. The laser diode power was set to 1 W and the size of the spot on the surface of the sample was about 2 mm. The results obtained for two different strands chosen on the surface of the sample ROS001 are presented here. A first PTE inspection was performed. Next, the plate was rotated 90° clockwise and a second PTE inspection was then performed. Inspections were expected to be also rotated 90° from each other.

**Figure 7:**
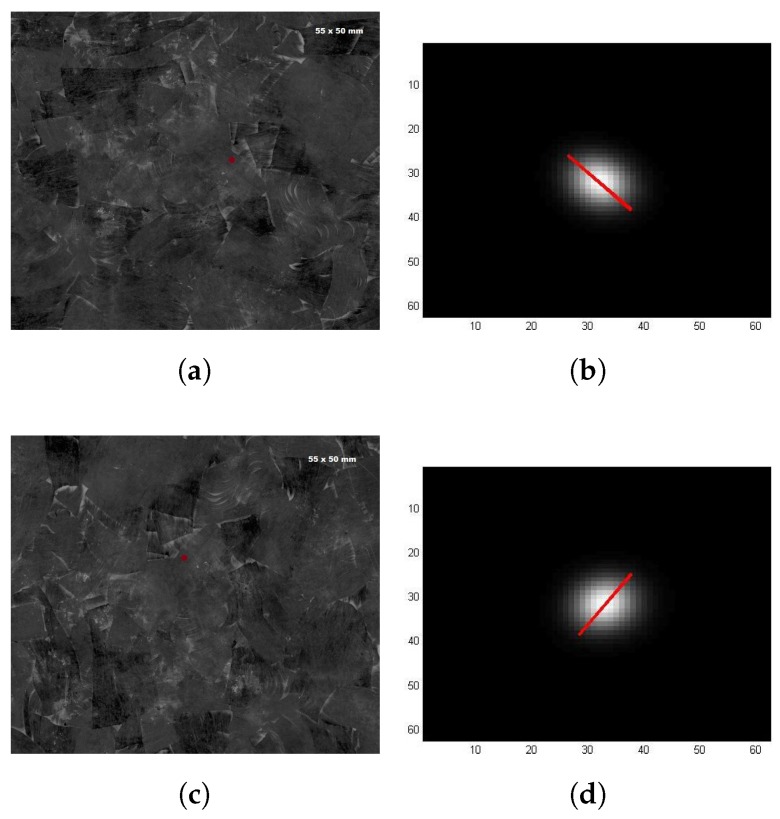
PTE inspection of single strand. First strand: (**a**,**b**) plate on its original position; and (**c**,**d**) plate rotated 90° clockwise.

**Figure 8:**
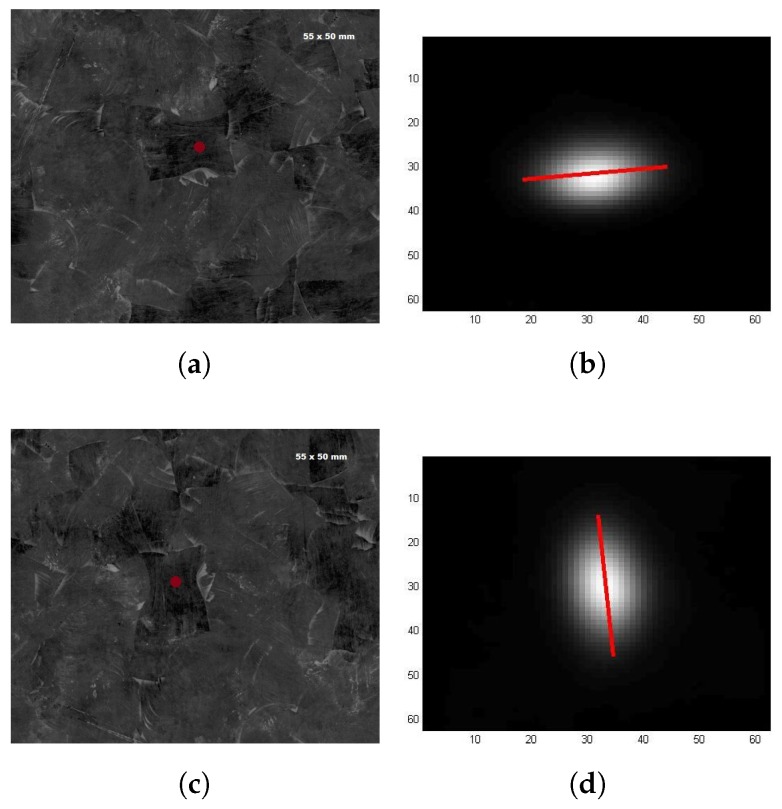
PTE inspection of single strand. Second strand: (**a**,**b**) plate on its original position; and (**c**,**d**) plate rotated 90° clockwise.

Figure 7 and Figure 8 show the results of those inspections. Figure 7 shows the results for the first inspected strand. Figure 7a,b shows the results in the case where the plate was in its original position, while Figure 7c,d shows the results in the case where the plate was rotated 90° clockwise. Figure 8 shows the results for the second inspected strand. Results for both strands are presented in a similar structure. Images on the left column display the picture of the region on the plate containing the specific inspected strand (position where the laser beam heated the surface is marked with a red point). Images on the right column display the respective ellipse segmented with the approach described in Section 2.1.1 (the red line indicates the ellipse’s major axis).

Fiber orientation measured in Figure 7b was −47.52° while the fiber orientation measured in Figure 7d was 55.99°. The ellipse extracted from the second inspection is then 76.49° shifted when it should be 90°. Thus, an error of approximately 13.5° is present in this case. For the second strand, fiber orientation measured in Figure 8b was 6.37° while the fiber orientation measured in Figure 8d was −85.19°. The ellipse extracted from the second inspection is then 88.5° shifted when it should be 90°. Thus, an error of approximately 1.5° is present in this second case. It is important to remember that all fiber orientation angles are reported regarding the x-axis.

For both strands, the difference between the two major axis measurements (fiber orientation), i.e., first when the plate was in its original position and then in when it is rotated 90° clockwise, should be 90°. However, it is not. For the first strand, the error between the two measurements was approximately 13.5°. This could be partially explained by the fact that the position of the heated area is not precisely the same in each case and also, in the first inspection a place nearer to the chip border was heated and it got more influence from a neighbor strand, which could have different fiber orientation. Additionally, during the plate’s moulding process heat and pressure are applied on the strands, which leads to a high degree of deformation in the shape of consolidate strands in the final plate and it could give to strands a high level of “shape interaction”. Thus, in some cases, neighbors strands can play a bigger role in the heating diffusion on a single strand, which could affect the elliptical pattern. For the second inspected strand, the error between the two inspections was approximately 1.5°. Here, the error between measurements is much smaller. This happened because the second strand is better placed and large enough so the heating spot could hit the same position with minimal edge or neighbor strands effects.

Therefore, it can be concluded from the computational results and this first experimental results presented that, similarly to the laminate case (which were approached in our previous works [16,35]), where the fiber orientation on each layer of the laminate is uniform, fiber orientation can also be assessed for ROS material using PTE.

### 3.2. Line Approach Results

**Figure 9:**
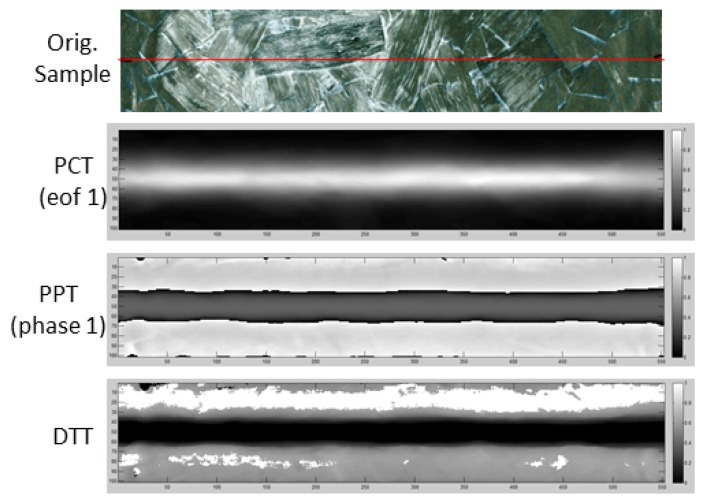
Results obtained with Principal Component Thermography (PCT), Pulsed Phase Thermography (PPT) and Dynamic Thermal Tomography (DTT) applied on the pseudo-static line.

Notwithstanding, the main goal of this work is to develop an approach that could estimate the fiber orientation on the surface of a ROS sample using a line heating region, which, in this case, was obtained with a flying laser spot inspection. In this sense, ANNs were chosen because they have the capability of estimating or approximating functions that can depend on a large number of inputs and are generally unknown, which is the present case. By analyzing the result obtained with the PCT, PPT and DTT application on the reconstructed pseudo-static sequence (see Figure 9), it seems evident that some of this information may be linked to the fiber orientation on the edges of the line envelope. However, a relation between this information and the actual fiber orientation is far from obvious. Thus, we propose to use an ANN.

The first step to employ an ANN to approximate this problem is to structure the network. Section 2.3 summarizes the ANN used in this work. Next, the network must be trained, i.e., the network must learn how to classify a sample. To train an ANN, a set of samples for which the classification of each sample is known beforehand must be created. In order to create a training dataset, 49 points were previously inspected by PTE on the same regions (49 on each region) where each flying laser spot inspection would be later conducted. The same approach used in Section 2.1 was employed to assess the fiber orientation of a single point using a static laser spot heating source. Thus, 588 PTE inspections, on 12 lines from three different samples were performed to create a database with known orientations that were used as ground truth. From this database, 412 samples were used to train the network. They were presented to the network as well as their respective classes obtained from the static single point inspections. During the training process, the network adjusts itself (its internal weights) to recognize the input samples presented during training into their classes with an acceptable error. From the other 176 samples, half were used for validation purposes. The other half was not presented to the network during training process and is used in order to test the network after training.

The pseudo-static sequences were processed with three different techniques: PCT, PPT and DTT. Thus, the training, validation and testing processes of the network were performed three times. Results were compared in order to select the best infrared image processing technique suitable for this specific problem. However, before the technique was applied on ROS samples, it was first tested on the laminate case. Recognizing the fiber orientation on the surface of a laminate sample is a trivial task and the results obtained with the ANN for all three stages, i.e., training, validating and testing, should be 100%, which was the case for all three infrared image processing techniques tested. After this previous step, the technique was applied on ROS samples where it would be much more difficult for the ANN to assess the fiber orientation. Results for the ROS case are presented in Table 1.

**Figure 10:**
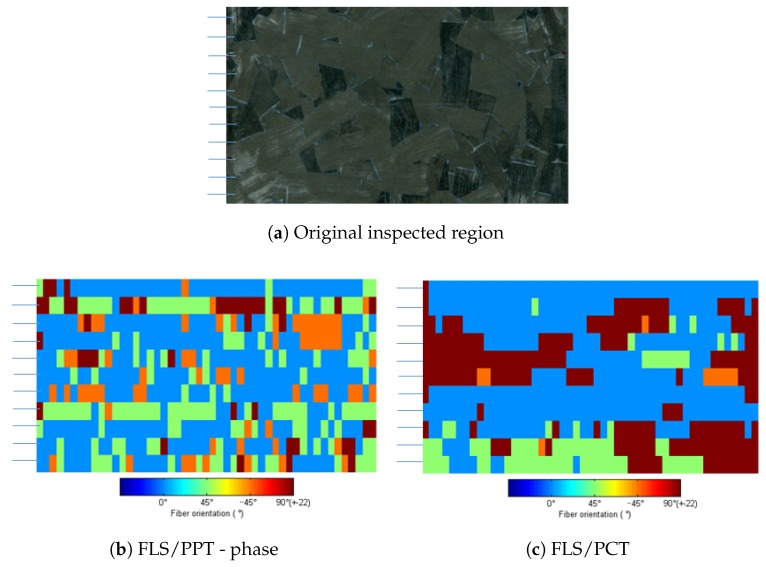
Classification results of fiber orientation obtained with samples not used during the training stage for the best image processing techniques (PCT and PPT). Results are color coded according to the color used in Figure 5.

A comparison of classification results over the same region is presented in Figure 10 for the two techniques that presented better results, i.e., PCT and PPT. The inspected region is 100 × 50 mm divided in 11 lines. The width of each line was about 1 mm and there was a 2 mm gap between each line, thus, the lines did not overlap. Finally, each line was divided into 49 samples. Results are color-coded according to class assigned to the sample at the ANN’s output. As can be observed, results from PPT are quite different from those obtained by PCT. Nevertheless, these results obtained are in coherence to the accuracy obtained in the test phase, i.e., for every four samples one was misclassified into another class.

As can be seen in Figure 10b,c, results obtained from PCT and PPT appear to be quite different. However, it can be observed that the differences usually occurred between neighboring classes and at this stage of the algorithm it is acceptable since each class covers 45° (±22°) and a sample which is placed near the border of one class could easily misclassified as the neighbor class. For example, a sample which has fibers oriented at 20° could be classified in class 1 (centered at 0°) by PCT and classified in class 2 (centered at 45°) by PPT since each class covers ±22° and the sample originally oriented at 20°.

The proposed approach only assess the fiber orientation on the surface of the ROS sample. However, as was done in [35], when fiber orientation through thickness was measured for a laminate moulded with continuous fibers, the approach proposed here could be adjusted to measure fiber orientation on deeper layers as well by adjusting the heating modulation frequency. At this point, it could be said that since the orientation at the surface has a quasi-isotropic behavior the distribution through thickness should have as well due to ROS manufacturing process.

**Figure 11:**
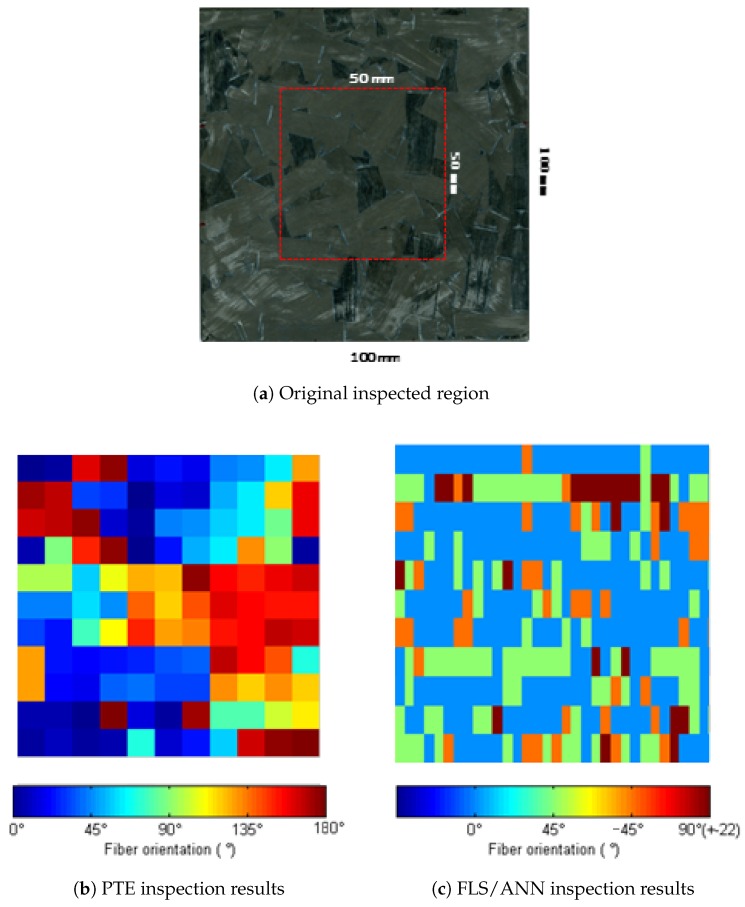
Comparison of point-by-point PTE inspection and the proposed FLS/ANN approach. (**a**) The inspected ROS region, (**b**) Results obtained with PTE inspections and (**c**) results calculated with the ANN for the FLS inspection for the same region.

Additionally, a 50 × 50 mm region was inspected with the static spot heating PTE technique and with the FLS/ANN technique. These results are compared in Figure 11. The image on the first line shows a picture of the inspected sample with the 50 × 50 mm inspected region depicted. The second image shows the result of inspection of the region with the point-by-point PTE inspection where 121 points were inspected (11 × 11 points). This inspection last about 25 min. Results are presented and color-coded in a range of 0° to 180°. The last image shows the inspection results of the same region but with the FLS/ANN approach. In this last case, 11 lines with 49 samples on each line are reported, i.e., 11 line inspections were performed with the FLS technique. This inspection took less than 30 s, which is great improvement compared to inspection time of the previous PTE inspection. Results are displayed color-coded according to the ANN output classes, as discussed in Section 2.3. The image processing technique used in this case was the PPT.

## 4. Conclusions

In this work, we presented an approach to assess fiber orientation of randomly-oriented strands samples. The technique is based on the classical thermal ellipsometry approach, here called pulsed thermal ellipsometry (PTE), which uses a single heating spot source to heat the surface of the sample while recording the thermal pattern formed on its surface. If the pattern is an ellipse, the material has anisotropic thermal properties and the ellipses major axis has the same orientation as the materials fiber direction.

Our proposed approach uses a flying laser spot (FLS) to heat the sample over a line region. Then, the thermal sequence is rearranged and processed with three different image processing techniques: PCT, PPT and DTT. From these processed images, features are extracted and an ANN is used to recognize the fiber orientation linked to that sample. Training and testing results are presented in Table 1. The best technique was PCT which presented an accuracy in the training stage of 91.3% and 71.6% in the testing.

Even though the results obtained with the ANN are not spectacular, the advantage of the proposed approach is the inspection time. For example, the inspection of the 100 × 11 mm long line reported in Figure 11 took about 25 min with the classical PTE inspection (inspecting point-by-point) while using the proposed approach the same region was inspected in under 30 s. This is an important factor to take into account when we want to apply a technique in the industry.

However, the number of classes recognized by the ANN is limited now to four in order to develop a first solution that could be improved afterwards. In the current state of this research, each class is 45° wide. However, if six or nine classes were used instead of four (30° or 20° wide, respectively, instead of 45°), the performance of the network would be severely affected in the current stage of development of this solution. Generally, the classification rate in the testing dataset decreased to 30% and 25%, respectively. Nevertheless, these first results obtained with four classes are very promising since they are being used as a start point for an improved approach.

## Figures and Tables

**Table 1:** ANN classification rates.

Input Image	Training	Validation	Test
PCT	91.3%	68.2%	71.6%
PTT	88.6%	55.7%	71.6%
DTT	80.6%	61.4%	71.6%
